# Czechoslovakia’s Cold War Arms Towns as Sites of Military Globalization

**DOI:** 10.1177/08883254261449260

**Published:** 2026-05-21

**Authors:** Rosamund Johnston

**Affiliations:** 1 University of Vienna, Austria

**Keywords:** cold war, Czechoslovakia, Slovakia, weapons, tanks, T-72, globalization, oral history

## Abstract

War has often been posited as the antithesis of globalization. This article, however, follows historian Paul Chamberlin in considering how the Cold War might be viewed instead as a moment in which new global linkages, revolving around “flows of arms, capital, technical knowledge, soldiers, and political ideologies,” were cemented. Focusing on the town of Martin—the biggest producer of tanks in socialist Czechoslovakia—it shows how military production simultaneously integrated the town and its citizens into transnational financial and intellectual networks and explicitly anti-Western alliances such as the Warsaw Pact. It pinpoints how exactly international linkages shaped processes of tank production, the experiences of tank producers, and indeed, the tanks produced themselves. A study of “military globalization” in Martin between the 1950s until the late 1980s reveals a complex patchwork of new openings and closings, as well as how enmeshed Eastern Europeans’ Cold War experiences were with the hot wars that “weaponized” the conflict outside Europe. It ultimately lays bare the role that military industries have historically played in processes of globalization, both within socialist Eastern Europe and beyond.

According to one socialist-era Hungarian joke, a man works at a fridge factory and decides that he too would like one for himself. So he starts pilfering parts in order to make his own fridge at home. But no matter which way he goes about assembling it, he laments to a friend, he always ends up with a tank.^1^

This joke reflects the widely held belief, on the part of those who told it, that communist economies were much more militarized than officially admitted. It articulates moreover a discontent with just how militarized—to the detriment of consumer goods—communist economies appeared to be. The joke demonstrates neatly how, as Arjun Appadurai claims, “the customary consumption logics of small communities are intimately tied to larger regimes of value defined by large scale polities.”^2^ Here the humor resides in a clash between those values: what the “large-scale polity” attributes worth to is completely useless for the inhabitant of this small community and his/her consumption logics. Rather than one set of values negating the other, however, I propose that we read this joke as testament to the different, sometimes contradictory, tracks of value that could simultaneously be present in a socialist arms town. In fact, it encapsulates the frictions inherent in the military globalization experienced in socialist Eastern Europe during the Cold War.

If the fridge-maker was voicing his disgruntlement with the predominance of military goods over civilian ones in the socialist economy, then historians have often focused on civilian goods rather than military ones to understand the global linkages of the economies in question.^3^ This article is inspired by the existing literature on socialist globalization, to be sure, but argues that acknowledging the role of military industries in socialist globalization has important implications for where we look for it and when. I agree with scholars who argue for the embeddedness of socialist economies within global financial systems long before the 1970s and would trace this embeddedness to lines of credit extended through arms deals (such as the Czechoslovak-Egyptian one in 1955).^4^ If works examining the cultural and political aspects of socialist globalization have moreover frequently focused on sites of higher education and administrative centers to understand its contours, then a study of the military aspects of socialist globalization takes the Eastern Bloc’s arms towns—often in remote areas and often wrongly assumed to be closed-off places—as its hubs or melting pots. I focus here on one arms town in socialist Eastern Europe (Martin, in today’s Slovakia), but I want to stress that the contribution made by military industries to globalization processes is no socialist aberration. Instead, I contribute here to a promising strand of literature arguing that war was more of a catalyst of globalization than has previously been recognized.

In what follows, I will take apart and reconstruct, above all, the weapon for which Martin is best known, the T-72 tank, to understand better the linkages connecting this site of its production to the rest of the world. Looking through the viewfinder of the T-72, one gains a clearer focus on both the nature of Soviet-“satellite” state relations and Czechoslovak citizens’ global engagements during the Cold War. My focus highlights the complex relationship between producers in Martin and the tank’s license-holder, the Soviet Union, underscoring that the experience of production was not just a case of the Soviet Union in miniature. Indeed, if the rollout of this tank across the Warsaw Pact constituted an attempt at military standardization, it ended up simultaneously creating and reinforcing difference in the spheres and sites of its production.

Instead of flattening or eradicating difference, military production in Martin simultaneously fostered, strained, prohibited, and reworked multiple international alliances—including with workers allegedly making tanks but wanting fridges in Hungary. Moreover, the global links resulting from military production simultaneously embedded towns such as Martin and their citizens in transnational financial and intellectual networks and avowedly anti-Western alliances such as the Warsaw Pact.

This article brings together the existing literatures on war and globalization on the one hand, and socialist globalization on the other, pointing out how each might enrich the other. It then sketches the broader geographies of Cold War trade and military production into which Czechoslovakia’s arms towns fit. Next, I turn to this article’s central case study, Martin, explaining what made it an “arms town” and the limits to understanding it as such. The sale of items produced at Závody ťažkého strojárstva (ZŤS)—a manufacturer of both military and civilian goods which became the town’s major employer during the socialist period—linked this town, through its products, to the rest of the world. I explore how these international linkages shaped processes of tank production, the experiences of tank producers, and indeed, the tanks produced themselves. Finally, I show how a complex account of military globalization processes present in socialist-era Martin cannot simply be reduced to an analysis of transactions taking place there in *valuta* (foreign currency), no matter how highly prized this came to be. In fact, the frictions caused by the forms of globalization that military production ushered in stemmed from the presence of multiple, sometimes contradictory, value systems operating alongside each other, occasionally overlapping and clashing in this socialist arms town.

These frictions become clearest when one juxtaposes Czechoslovak Politburo and Warsaw Pact sources with factory newspapers and oral histories recorded with inhabitants of Czechoslovakia’s arms towns. Rather than privileging one set of sources over the other, I suggest that the comparison of texts produced within “small communities” and “large-scale polities” illuminates the complexity of military globalization.

Oral histories grant a privileged window onto military globalization as experienced and constructed “from below.” They reveal more articulately than any Politburo documents or police reports some of the frictions that emerged when large export contracts or sizeable numbers of foreign laborers arrived in these arms towns. I draw here from a corpus of twenty-six interviews that I recorded between 2020 and 2026 with those working in the military and civilian manufacturing sectors in Czechoslovakia’s arms towns. I used the snowball method, often making initial contact with former workers who had written about their town’s arms manufacturing history in the media. More men have thus far agreed to be interviewed than women, although women regularly made up at least one half of the workforce producing arms. In addition, more white-collar workers (engineers with a degree from a technical college or administrative staff in these factories’ planning or sales divisions) have thus far agreed to an interview than manual workers. Social mobility in communist Czechoslovakia’s arms plants, however, meant that several of these office workers began as manual workers. Neither are the views of lifelong blue-collar workers absent from this sample, which continues to grow, as this research is still ongoing.

Factory newspapers like the ZŤS weekly *Turčiansky strojár* (*The Turiec Engineer*) shed additional light on military globalization at the local level. Such publications could at once be mouthpieces for the communist regime and highly articulate windows into the subjectivities of those who authored them, as the worker–journalists who contributed to them make clear.^5^ While acknowledging the lack of explicit discussion of arms production and export, as well as the publications’ centralized, syndicated content, I am inspired by historians of socialist Yugoslavia, Goran Musić and Anna Calori, to argue for their value as readily available sources shedding light on the weekly rhythms, accords, and discords of the factories and factory towns in which they were based from the 1950s up until the 1990s.^6^

I further compare these newspapers with government documents which put the labor and commodity exchanges inflecting the lives of citizens of Czechoslovakia’s arms towns in a broader economic and geopolitical perspective. For all their frankly questionable figures and highly euphemistic language when referring to arms, Politburo documents offer historians a useful starting point if they wish to “follow the money.” Lastly, this article incorporates ZŤS corporate files to reveal the multiple vectors of military globalization cohabiting in Czechoslovakia’s arms towns.^7^

## The Case for “Military Globalization”

War has often been posited as the antithesis of globalization, with a large body of literature dating the end of the first wave of globalization to the beginning of World War I.^8^ But Adam Tooze and Michael Geyer have convincingly argued against this view, taking World War II as their example. They posit instead that the “World” war was won precisely on account of the ability of one of the belligerents to turn the conflict into a global clash and to sustain it globally.^9^ Following their lead, some scholars have begun, indeed, to posit the international arms trade as a factor *shaping* the global order of the early twentieth century.^10^

It was the Czechoslovak arms trade, estimated by the Politburo to make up nearly 15 percent of the country’s total manufacturing, that anchored the country into transnational networks such the Warsaw Pact and Comecon on the one hand, and United Nations conventions (for example on plastic explosives) on the other during the Cold War.^11^ For historian Paul Chamberlin, the Cold War cemented new transnational linkages, which revolved around “flows of arms, capital, technical knowledge, soldiers, and political ideologies.”^12^ Peter Švik and Wolfgang Mueller argue that warfare in the second half of the twentieth century “fanned not only technological innovations but also their transfer,” which took place through licit and illicit sales, as well as processes of gifting, intellectual theft, and seizure on the battlefield by combatants themselves.^13^

Likewise, Tarak Barkawi urges his reader to “think about how different localities come to be connected and shape each other” through conflict.^14^ He argues that arms races, such as those undertaken by the superpowers in concert with their allies during the Cold War, should be viewed as “one form of connection between societies.”^15^ Certainly, if the arms production and stockpiling of the Cold War pitted East against West rhetorically and closed possibilities for the mobility of certain people and goods, then they also fostered a drive to understand the activities of one’s rivals, leading, for example, to the deployment of American spy technology over the hills and valleys of Western Slovakia.^16^

Barkawi focuses primarily on soldiers on deployment and armies, highlighting the extent to which globalization processes might be mediated by individuals and their own lived experiences. I focus instead here on the Cold War’s “home front” to stress how enmeshed Central Europeans’ Cold War experiences were with the hot wars that allegedly “weaponized” this conflict outside Europe. At the same time, a focus on the supply chains and economic linkages binding Czechoslovakia’s arms towns into the global Cold War can change our received geographies and chronologies of globalization in Eastern Europe.

Rather than a primarily economic process, globalization for Barkawi is a question of consciousness (which is intimately linked to one’s economic standing). His view reinforces Angelika Epple’s definition of globalization as “an asymmetrical, pluralistic, non-linear, non-teleological and multilayered process of entanglement taking place at different speeds, which is pushed forward, slowed down, transformed and altered by individual and collective actors.”^17^ Her definition provides the basis for the analysis of globalization undertaken here.

Barkawi ultimately equates globalization with “westernization,” arguing “there is a strong but neglected military dimension to processes of worldwide westernization that have so occupied scholars of imperialism and contemporary globalization.”^18^ Scholars of socialist globalization have rightly pointed out that accounts such as this which bring the First World into conversation with the Third World, using the latter as a means of unseating the “Eurocentric notions” captured in the former, can at times oversimplify “core-periphery” dichotomies.^19^ Such accounts of globalization which fail to take the socialist world seriously run the risk of overlooking the experiences of “roughly one third of the world’s population” who inhabited “the socialist world at the height of its expansion around 1980,” a serious oversight for a literature that is claiming to chart shifts on a global scale.^20^

But in failing to acknowledge its strong military components, the literature on socialist globalization has likewise hitherto overlooked some of its important venues and actors—and this omission can in fact produce erroneous conclusions. Oscar Sanchez-Sibony, for example, correctly argues that it was the norm, not the exception, for Soviet and Eastern Bloc economies to be embedded in global economic structures during their time as socialist states.^21^ But he expressly states that he is exempting the foreign trade in military materials from his argument. This means, however, that he is only half able to describe, for example, the nature of the relationship between the Eastern Bloc and Egypt “from Stalin to Khrushchev,” which certainly turned on cotton, as he suggests; yet, as military contracts housed in Prague show, small arms, munitions, and tanks from Martin accompany the credit lines extended to Cairo that provided the basis for such cotton shipments in the first place.^22^ Sanchez-Sibony productively reframes the chronology of socialist globalization, suggesting it started earlier than the 1970s. The picture he presents would be enhanced further by acknowledging the extent to which military materials were the axis on which this embedding often turned.

**Figure 1.**
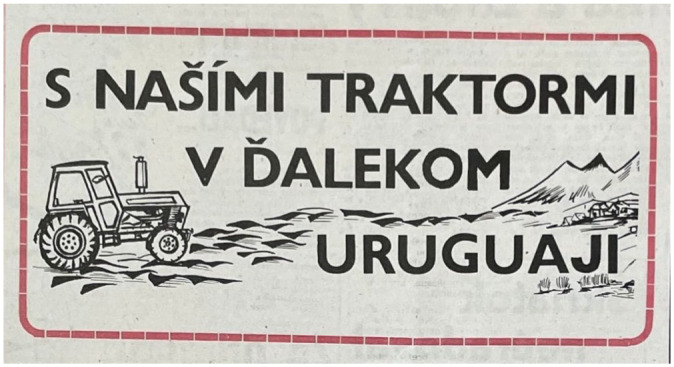
Illustration highlighting the presence of ZŤS Martin’s civilian products (Zetor tractors) in “faraway” Uruguay—then a U.S.-supported military dictatorship. Source: Slovak National Library. *Turčiansky strojár* 31, nos. 35–36 (16 September 1981): 6.

It is fitting that recent scholarship has rejected the equally erroneous notion that all Eastern Bloc international relations were based at root on force, violence, or “hard power.” However, we stand to grasp the complexity and the numerous institutions invested in processes of socialist globalization better by acknowledging that military cooperation could, and often did, pave the way for other interconnections. If Czechoslovak-Egyptian relations began through military exchange, to pursue the example given earlier, then it can be inferred that they led to the blossoming of Egyptology as a discipline in Czechoslovakia, generating its own form of “humanist” expertise and intellectual infrastructure (in the form of the Egyptological Institute in Prague, founded three years after the Czechoslovak–Egyptian arms deal).

Neither were military and development ties an either/or proposition. As the joke that opened this article acerbically notes, the military and the civilian—the tank and the fridge—were often inextricably linked at their point of production. This is not to say that those working in the civilian branches of a factory could negotiate military sales on their dispatch abroad as well: they could not, as the foreign trade in military and civilian materials was neatly divided between separate organizations in socialist Eastern Europe. But their interlinking at the point of manufacture has implications for how we understand the economic fortunes of behemoth producers such as ZŤS whose revenue was generated through a combination of swords and plowshares.

Accompanying the tanks that left ZŤS Martin’s production line were tractors, diggers, and locomotives.^23^ The trade in civilian and military goods could go hand in hand (as was the case in Egypt) but could also complement one another, following completely different circuits. *Turčiansky strojár* trumpeted, for example, how the plant’s civilian vehicles were sold to avowedly anticommunist states where Czechoslovak weapons were much less likely to be found.

Weapons production peaked in Slovakia in the late 1980s. At ZŤS Martin, historians Jan Štaigl, Peter Turza, and Dušan Mihálik estimate that 64 percent of the workforce was employed in military manufacturing.^24^ Those directly employed by the arms industry across Slovakia had grown by 50 percent over the previous two decades, and weapons made up almost a quarter of all Slovak machine manufacturing.^25^

The rise in arms production correlated with a rise in arms exports. Yet the rich recent scholarship on socialist globalization points in the opposite direction to the picture of ever-intensifying military globalization drawn here. Béla Tomka attributes this to the existing literature’s disproportionate focus on “political and cultural” ties.^26^ The authors of such works can, indeed, be reflexive about this. For example, *1989: A Global History of Eastern Europe*—a work reframing the significance of that year in Eastern Europe and the region’s relations with the rest of the world—admits that the region’s military industries defied the trend of “declining globalization” detected in other spheres.^27^ Indeed, it is hard to argue for the “deglobalization” of military production in 1980s Czechoslovakia: the U.S. State Department estimated that Czechoslovakia ranked among the top ten global arms exporters annually throughout the 1980s, with weapons making up a bigger share of the country’s total exports by 1989 than ever before.^28^ Using official Czechoslovak sources, Štaigl and Turza claim that Slovakia became the “third biggest maker of armored vehicles in the world” during the 1980s, trailing only the two superpowers.^29^ The venues for military deliveries were more widespread than ever, encompassing Algeria, Iraq, Syria, Libya, and all Warsaw Pact states for products made in Martin alone (see Table 1). Economist Yudit Kiss attributes the upswing in Slovak military production during the 1980s to the Iran–Iraq war, which led to the “blossoming” of arms production, centered in particular in and around Martin.^30^

**Table 1.** Exports of ZŤS Martin Tanks, 1951–1991.

	T-34	T-54	T-55	T-72
Czechoslovakia	706	2,249	2,193	864
Bulgaria	238			92
Romania	335		135	
Egypt	406	202	430	
Hungary	100	76	564	46
East Germany			1,764	263
Yugoslavia			552	
India		225	99	
Morocco		79		
Libya			1,253	119
Syria		58	1,331	258 (of which 50 prior to 1989)
Unidentified			1	152
Iraq				90
Algeria				106

Source: Calculations based on the research of František Ježek and Vladimír Francev.^31^

Note: Numbers are estimations that include tanks and their variants.

## Geographies of Production and Trade

Czechoslovakia ranked behind the Soviet Union as the second largest arms producer within the Warsaw Pact. Indeed, Daniela Richterova, Mikuláš Pešta, and Natalia Telepneva argue that during the Cold War, Czechoslovakia’s sprawling arms industry brought it into direct competition with “more powerful players in the world market, including the Soviet Union.”^32^

Between 1949 and its demise in 1991, the Council of Mutual Economic Assistance (COMECON) sought to instill specialization of military production among member states. Initially, COMECON’s Soviet administrators looked to the Czechoslovak arms industry, which remained relatively intact after World War II, to help rearm the rest of the Eastern Bloc.^33^ Over the decades that followed, the Soviet generals and functionaries setting COMECON’s military production agenda sought to keep the manufacturing of hi-tech military goods (such as rockets and nuclear weapons) within the Soviet Union, delegating the production of “lower-tech” military goods (which were less shrouded in secrecy and therefore easier to export beyond the Warsaw Pact) to the Eastern European “satellites.”^34^ In theory, Hungary (which never made whole tanks, contrary to what the article’s initial joke may have suggested) was to specialize in communications equipment and military radios, Bulgaria became a major center of ammunition production and military computers in late socialism, while Poland produced tanks, warships, and other naval equipment. Czechoslovakia manufactured 85 percent of the entire portfolio of Warsaw Pact military equipment, calculated one employee of the then-state arms-export monopoly, Omnipol.^35^ An overlap thus did exist between the military goods that different Warsaw Pact member states produced. This in turn spurred the “competition” that Richterova, Pešta, and Telepneva detect between Eastern Bloc states selling similar products on the world market.^36^

Specialization across COMECON was supposed to result in each member state supplying the others with the parts that each required. The trade for parts among COMECON members took place in convertible rubles, but this boiled down to “trade in kind, thus no true cash movement attended the deliveries of either civilian goods or military equipment among [the member states].”^37^ At times, however, foreign trade officials eschewed convertible rubles altogether and undertook straightforward acts of barter, sometimes for non-military goods. “Exceptional” cases of barter characterized the negotiation and renegotiation of arms contracts beyond COMECON too. For example, Czechoslovakia asked Cuba for cigars when Havana was unable to repay its debts; foreign trade officials sought oil from Libya when it could no longer pay its bills in dollars, as this Omnipol salesman (who worked on the deal) recalls.^38^

Socialist Czechoslovakia was technologically and infrastructurally capable of executing arms deliveries by itself, finds Tomáš Nigrin, with river shipping capacity on the Danube and the Elbe, a maritime fleet, and a national airline with a dense network of flights to the state’s major trading partners.^39^ And yet Czechoslovak officials repeatedly asked for Soviet, Polish, and Yugoslav help to deliver arms.^40^ Politicians oscillated between using their own national carriers and those of other states: while the former taxed Czechoslovak coffers less, the latter heightened plausible deniability. Scholarship on a series of scandals that led government ministers to adjust transportation norms currently provides the best window onto how the shipment of Czechoslovak weapons worked.^41^ But by emphasizing the daily realities of arms transportation over and above these exceptional moments, Tomáš Nigrin’s research offers us an even richer understanding.^42^

The largest customers for Czechoslovak weapons were almost always to be found in the Middle East and North Africa (MENA). The arms deal that “put Prague on the map,” for example, was with Egypt in 1955.^43^ While greenlighted by the Soviet Union and containing Soviet weapons disguised as Czechoslovak ones, it paved the way for follow-up deals negotiated directly between Cairo and Prague (both to service existing equipment and provide more).^44^ As relations with Egypt cooled over the following two decades, Czechoslovak trade officials used their positions in Cairo to strike up contacts with what would become the next major client for Czechoslovak arms: Libya.^45^ According to Matyáš Borovský, Libya’s “main interest” in early talks held with Czechoslovak officials in the mid-1970s was the T-55 tank, constructed and modernized for export from the Slovak town of Martin.^46^

Military historian Igor Baka describes the period from the mid-1970s until the mid-1980s as the “peak” of Czechoslovak–Libyan cooperation.^47^ Libyans became the single biggest group of foreign students in Czechoslovakia, based at military academies and civilian institutions. Technicians from Czechoslovakia’s arms towns traveled to Tripoli, Sirte, and Benghazi to teach, establish a factory producing spare tank parts, and service the military equipment worth more than one billion U.S. dollars sold through Omnipol to Muammar Gaddafi’s regime.^48^

Through the trade first with Egypt and then with Libya, many Czechoslovak arms made their way around the “Global South,” the focus of this special section. First Gamal Abdel Nasser and then Gaddafi made no secret of their plans to use the weapons they purchased in socialist Eastern Europe to achieve their own geopolitical objectives.^49^ Indeed, Foreign Ministry files show that Czechoslovak policymakers were aware that the arms they were shipping were used elsewhere (a fact about which the CIA also held few illusions).^50^ Weapons from Egypt (alongside Czechoslovak arms donated by Algeria and Ghana to the Organization of African Unity) armed Biafran separatists in 1968 in what became an international scandal for Prague, which was exposed as arming both sides.^51^ One decade later, tanks retrofitted in Martin were sent by Gaddafi to fight “imperialist” French troops in Chad’s civil war.^52^ The presence and use of Czechoslovak arms in the Global South underscores that the global story of Czechoslovak arms was never one whose terms were dictated by Czechoslovak actors alone. In the rest of this article, I explore what these complex geographies of production, trade, and circulation meant for those inhabiting Czechoslovakia’s Cold War arms towns.

## Martin, a Czechoslovak Cold War Arms Town

Arms production took place across Czechoslovakia, but it increasingly came to be concentrated in the “military triangle” clustered around the Váh river in western Slovakia.^53^ One of the points on this triangle, the town of Martin became the biggest producer of tanks—and military equipment of any sort—in Czechoslovakia by the end of the Cold War. First specializing in T-34 tank production, the Martin plant then added the T-55, and then, over the course of the 1980s, the T-72 to its production portfolio. When they decided to wind down military production in 1989, government officials in Prague judged that Martin would be the worst-hit municipality in all of the country.^54^ It is in light of its nearing mono-industrial nature by this period that I call Martin an “arms town.” This was also the case with the other points on this “military triangle”: Detva and Dubnica, alongside Uherský Brod (a major producer of small arms) in Moravia, but not so with the municipality second worst affected by this conversion, Brno, which housed electronics and textile industries, as well as military production. As the bulk of Martin’s output was military, I would likewise argue that we should view the interconnections fostered within the town through, first and foremost, a military lens.

**Figure 2.**
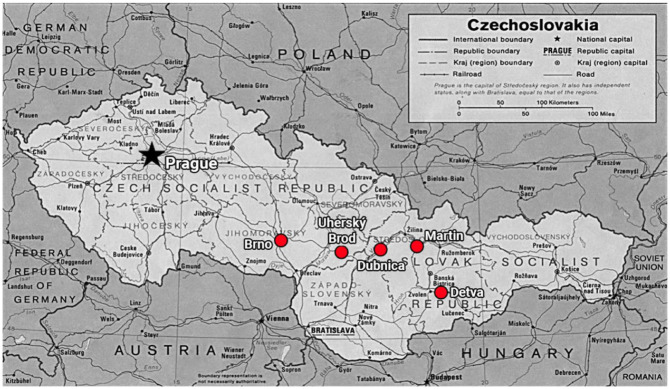
Map of socialist Czechoslovakia with major sites of arms production highlighted. Image: Tomáš Horalík.

Tank production had moved from Prague to Martin gradually after World War II. Jaroslav Láník notes the complications attending the initial rollout of production in Martin but shows how government ministers redoubled their efforts to increase capacity there—even during the late 1950s, which saw military spending cuts elsewhere in Czechoslovakia, as the armored vehicles to be produced in Martin were in critically short supply in the Czechoslovak military.^55^ Hand in hand with the expansion of production came a drive to create more of the production materials on site. Láník notes that Czechoslovak planners sought to produce 66 percent of cast steel and 90 percent of forged steel domestically by the late 1950s, expanding metallurgical capacity in Martin.^56^ While they boosted steel production in the arms towns, complaints about relying on deliveries from overseas never fully abated under socialism.^57^

**Figure 3.**
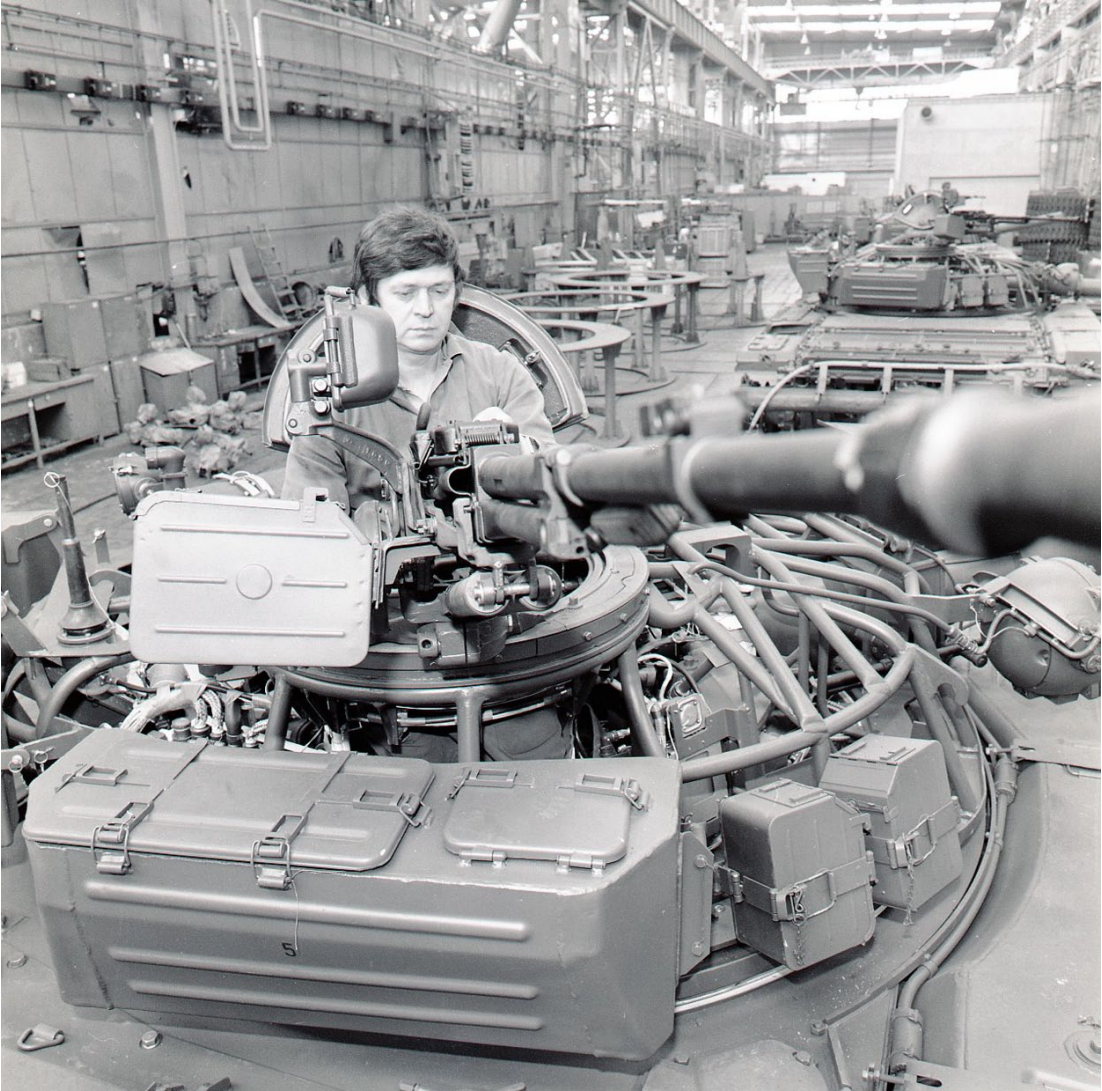
Worker assembling a tank at ZŤS Martin in 1991. Source: Tlačová agentúra Slovenskej republiky (TASR).

Furnaces from Austria to make this steel were tracked by the CIA arriving in the Czechoslovak arms towns in the 1950s.^58^ Later, workers in Martin recall machinery from the West German firm Siemens, purchased for use in civilian production and deployed to more military ends. But machines from West Germany and Austria were notably rare; more frequent was the development of production machinery in-house. ZŤS had both a large tooling department and an affiliated research institute in Nová Dubnica dedicated to technological development. Dušan Mihálik, whose father worked at ZŤS, notes that the frequent replacement of tools was a perk of working in the Czechoslovak arms sector.^59^

ZŤS Martin grew in political importance as its workforce grew. Following its establishment in 1948, municipal files show how the plant (at the time named after Soviet leader Joseph Stalin) frequently came second to the collectivization of agriculture on the agenda preoccupying politicians in this previously strongly agricultural region.^60^ The breakneck pace of growth at the factory meant that the population of Martin trebled to thirty-three thousand by 1961 and had transformed in its composition from peasant to industrial worker, with the arms plant now the region’s primary employer.^61^ At the peak of its expansion during the 1980s, ZŤS Martin employed more than twelve thousand workers.^62^

Regional Communist Party meeting notes show that the higher wages paid by the plant lured Martin inhabitants away from agricultural work.^63^ Vladimír Jančovič, an engineer, recalled how it attracted him and others working in civilian industries in nearby Žilina.^64^ On top of their “above-average wages,” arms workers enjoyed bonuses, explains Dušan Mihálik, for example, after having met production targets.^65^ As well as being drawn from other local industries and nearby towns, the ZŤS workforce, on some occasions, reproduced itself. Workplace romances led to some ZŤS families, with a second generation passing through the ZŤS nursery and then the training school attached to the plant, before taking a job in the factory.^66^

Laborers from overseas also boosted staff numbers at ZŤS Martin. Workers from Vietnam were employed from at least the mid-1970s, with ZŤS offering Vietnamese staff both training and practical experience working in heavy industry, the explicit wish of the Vietnamese government when establishing bilateral labor exchanges with Czechoslovakia.^67^ As the plant expanded in the 1980s, and Czechoslovak–Vietnamese relations became increasingly “beleaguered,” Vietnamese workers came to plug labor shortages.^68^ I found no official documents stating the number of Vietnamese employees at the plant, but factory directives housed at the Slovak National Archive outline ZŤS managers’ obligations toward their Vietnamese workforce.^69^ Furthermore, *Turčiansky strojár* repeatedly brought the presence of the Vietnamese laborers in their midst to Martin residents’ attention, for example, with profiles of Vietnamese traditions in the run-up to Chinese New Year, a holiday granted at the plant to Vietnamese laborers, but not their Czechoslovak peers.^70^

When Libya became the biggest customer for Czechoslovak weapons outside of the Warsaw Pact, Libyan workers arrived in Martin. They received less press coverage styling them as a “model minority” but equal attention from factory workers and town inhabitants in interviews.^71^ Igor Baka suggests that more than seven hundred and fifty Libyan citizens were supposed to be sent to Czechoslovakia in 1983 to study and gain practical experience in military production.^72^ An unknown number became resident in Martin. Matyáš Borovský judges the resultant intermingling of Libyan and Czechoslovak citizens not to have been “without conflict,” leaving a trail of “transgressions” documented by the secret security forces.^73^ Igor Baka agrees, stressing that run-ins with police and skirmishes between Libyans and Czechoslovak citizens had the effect of worsening bilateral relations.^74^

As Borovský and Baka make clear, workers’ solidarities with their confrères from the “developing world” (in the language of the time) were far from straightforward and themselves marked by contradictions. On the one hand, solidarity was solicited through occasional “solidarity shifts” or other work drives to create materials or raise funds for foreign causes, for example, in Vietnam.^75^ These perhaps did foster connections, constituting real investments of time and income on the part of ZŤS workers. But such solidarities were complex, and workers often labored to resolve the economic imperatives of such shifts with other sets of values. In Uherský Brod, for example, Zdenka Hodická accepted such intensive solidarity shifts as a part of her job but acknowledged her distaste for weapons and questioned their deployment to the very venues she supported through such arms-making drives.^76^

The presence of foreign nationals in the arms towns gave rise to new practices to maintain secrecy. A directive circulated among ZŤS employees in 1979 stressed that foreigners should be monitored as they moved around the factory.^77^ Employees were urged not to make too many copies of technical plans, to lock their offices and the filing cabinets which stored secret documents, and to school their colleagues in the political importance of discretion.^78^ That arms and armaments production were amongst the most sensitive of state secrets in socialist Czechoslovakia is reinforced by a somewhat counter-productive “list of secrets” circulated among factory management in 1984, whose very first page underscored that questions of defense and national security were not to be widely discussed.^79^ Interviews suggest that different members of the same family could enjoy different levels of security clearance (and therefore access to parts of the plant and documentation), with narrators presenting this as a given, rather than something they struggled to accommodate.^80^ As Dušan Mihálik reminds us, this secrecy worked at the international level to keep Czechoslovak engineers somewhat in the dark too: with technical details of military production sometimes not shared by Soviet license-holders, a certain amount of reinventing the wheel was required by workers in arms towns rolling out the production of new types of weapons.^81^

Military materials featured in everyday life in Martin beyond the sites in which they were produced. One of the Czechoslovak People’s Army’s tank divisions was stationed in town. Locals who were not privy to processes of tank construction explain that they knew when these soldiers were testing ZŤS tanks by the sounds of gunfire that resonated in the surrounding hills.^82^

*Turčiansky strojár* concluded that ZŤS expansion “was tightly connected to the development of Martin and the whole of the Turiec region.”^83^ The newspaper explained that the factory played an important role in the town’s layout as it built housing for workers. ZŤS also offered workers building materials and housing patterns to build their own homes, further shaping the look of the municipality. Beyond what the eye could see, the factory was responsible for some of the town’s infrastructure, such as the energy to power the winter stadium and its swimming pool, which managers procured through barter with the state-owned-enterprise *České loděnice* (whose original purpose was as a dry dock for repairing ships).^84^

Yudit Kiss stressed that factories like ZŤS set the rhythms of the arms towns, with bus timetables planned around their shifts.^85^ But lest this picture appears too all-encompassing, or too neat, there were two important limits to the extent to which arms production shaped Martin. On the one hand, production of civilian goods also employed families and fostered international connections between ZŤS and overseas buyers. On the other hand, the same *Turčiansky strojár* celebrating the factory’s impact on the town contained polemics aimed at inhabitants decrying their lack of engagement in extra-curricular activities organized by factory agitators allegedly on their behalf. In one example, workers were harangued when a lecture on the lives of their brethren in socialist Mongolia was met with an embarrassingly low turnout.^86^ For all the attempts to build solidarities and commandeer aspects of their free time, Martin’s inhabitants had their own ideas about what leisure might look like, which were sometimes at odds with factory-spearheaded initiatives to channel this.

**Figure 4.**
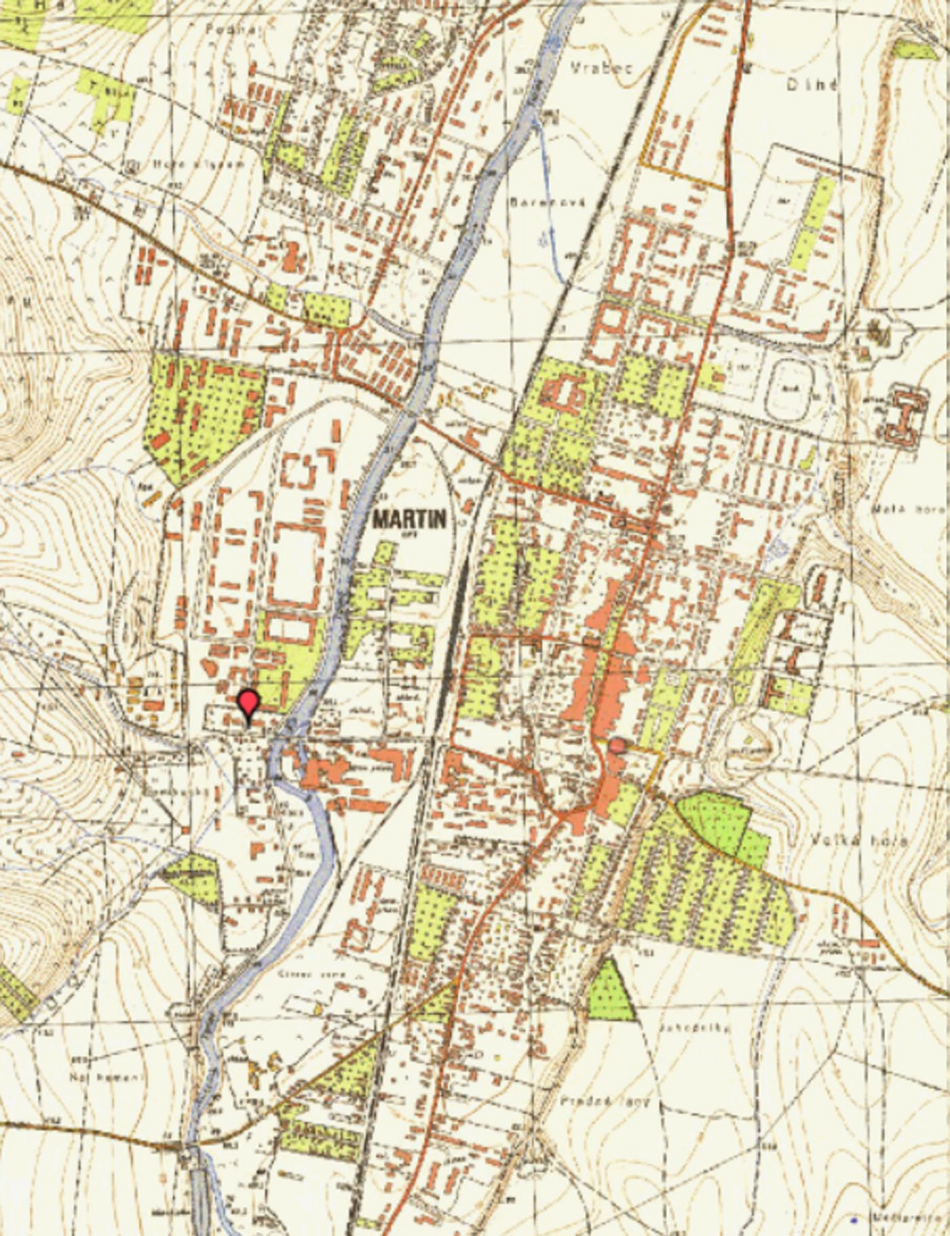
Map of Martin, 1964, reflecting the town’s expansion and practices of secrecy. The arms plant (on the left of the river) is barely outlined, and the tank brigade’s barracks (in the top right quartile) are almost absent. Source: Staremapy.sk.

## Relations Forged through Trade

In the Czechoslovak socialist economy, weapons production and foreign trade were separated, and therefore, producers in Martin did not broker deals directly with foreign clients (this was done instead by employees of the foreign trade enterprise Omnipol—based in Prague, but with one permanent representative at the ZŤS plant). Yet producers’ dispatch, and the dispatch of their colleagues, overseas on service contracts—coupled with the presence of workers from allied states alongside them in ZŤS—left little doubt as to where their products were going, and to what effect. In Appadurian terms, Martin and its inhabitants were far from “enclaved” in their work producing arms. This is evidenced by the few myths uttered by producers on the topic of where their handiwork ended up and the use to which it was put.^87^

In fact, in oral histories, inhabitants of Slovakia’s “military triangle” at times contrast their allegedly superior knowledge with the supposed naivete of Prague-based political liberals following the revolution.^88^ Such accounts seek to discredit policy decisions made to pursue further arms industry conversion in the 1990s on the basis of their supposedly weak intellectual foundations. But they also provocatively invert the idea of periphery and metropole when it came to Czechoslovak arms production and the international connections its fostered, casting sites of production as knowledge centers and sites of arms administration as provincial backwaters.

Factory newspapers reveal both the sorts of relationships that were forged through the international trade in ZŤS products and the limits to such connectivity, socialist style. If it declared itself disappointed in readers for their disinterest in the official expressions of socialist internationalism organized for them to attend, *Turčiansky strojár* also presented its audiences with a succession of VIP delegations from Cuba, Vietnam, and the Soviet Union gracing their workplace.^89^ It referred to the products manufactured at ZŤS as going “out into the world.”^90^ Articles often identified the destinations to which civilian goods were traveling (but never explicitly mentioned military goods), with accounts of ZŤS-produced forestry machinery in China, or interviews with the firm’s engineers upon return from Cuba or Vietnam.^91^ When referring to broader trends, reporters at the ZŤS factory newspaper championed a rise in exports, not multidirectional connectivity in and of itself: their focus on exports suggests that factory journalists strongly prioritized the travel of goods and experts in one direction over the other.^92^

Military trade took place simultaneously along several, inconvertible, economic tracks: barter was one of these, alongside the use of Czechoslovak crowns and convertible rubles for the trade in components.^93^ The vehicles assembled at the ZŤS plant were then often sold overseas beyond the Warsaw Pact for hard currency.

Martin’s flagship product, the T-72 tank, opened new possibilities for international linkages, but closed some existing ones too. For a time, the tank’s Soviet license-holders prohibited the sale of this newer model to Prague’s biggest clients beyond the Eastern bloc (in a standard move on the part of a weapon’s license owner rather than an egregious case of Czechoslovak victimization by a Soviet overlord, as retrospective accounts can suggest). Miroslav Belica, a trade attaché in Libya in the 1980s and the author of one such account, judged the creation of the T-72 tank as a bad move for the Czechoslovak arms industry:

we added a stronger motor and also more resilient, lighter armor to the Soviet T-72 tank, made from an alloy that we had developed. But the Soviets wanted these modernized tanks all to themselves, and didn’t allow them to be exported.^94^

In his view, the Soviet Union used license agreements as a lever to control arms exports. He qualifies this statement in practically the next line, however, suggesting that bribes could subvert the official Soviet export policy, a move he grumbles that the East Germans were more adept at employing, allegedly, than he and his Czechoslovak colleagues.^95^

In such circumstances, a culture of repurposing and salvage became an economic imperative for the ZŤS plant. The vast majority of the factory’s tank sales for hard currency during the 1970s and 1980s—to Libya, India, Syria, and Egypt—were of renovated older models, notably the T-54 and T-55 (Table 1). Over one thousand two hundred of these were sent from Martin to Libya between 1971 and 1981.^96^ Soviet officials urged their Czechoslovak counterparts to consider winding down work repairing older T-55 tanks, but this did not happen as this trade in now-defunct weaponry was lucrative both for ZŤS and Omnipol staff.^97^

Technicians in the Czechoslovak arms towns tinkered with the models they produced under Soviet license. Martin engineers took their adaptation work so far that they created variations of a T-72 officially codified as such. This redrew the rules of the new model’s intellectual ownership and sale. ZŤS staff developed and mass produced altered, T-72MK and T-72M1K command tanks, with enhanced radio and navigation systems, which were then sold to East Germany, Hungary, and in the late 1980s, Algeria.^98^ By that time, the T-72 became available for sale beyond the Warsaw Pact, to Libya, Iraq, and Syria.

Martin boasted a Tuzex store which sold Western goods for Western currency—normally the preserve of much larger towns in Czechoslovakia—on account of the sheer amount of hard currency there, possessed by the factory’s local employees who traveled the world to service the factory’s goods and the foreign laborers who came to work at ZŤS. *Bony* (vouchers that could be used in this store purchased with Western currency) created their own social hierarchies within ZŤS, with some factory employees being known to have a surplus to trade.^99^ In what sounds like an urban legend, narrators insist (vaguely, but evocatively) that the amounts of foreign currency present in the town made the place a hub for gold-digging female visitors “from as far away as Moravia.”^100^ Regardless of their meaning and accuracy, such statements invoke female sexuality to amplify narrators’ conviction that these arms towns became regional economic centers.

Business trips overseas also allowed those who undertook them the chance to acquire consumer items that were not widely available in Czechoslovakia at the time. ZŤS technician Pavel Kotrik voiced his pride at acquiring jeans and Walkman for his family, which bolstered his relatives’ standing within Martin (where, for all the presence of East Asian consumer electronics and Western fashions, they were still rare enough throughout the 1980s to confer significant social prestige).^101^

## Relations Forged through Production

The mass production of the T-72 tank was paradoxical from its start in 1981. It bound Martin into ever closer cooperation with similar arms towns in neighboring states, but created discontents, and even the impetus for increased autonomy in certain fields. And if the T-72 was produced according to a “Soviet model,” then deviation from and “improvement” on this model could prove a source of pride for Martin inhabitants. The process of T-72 production reveals the complexity of Czechoslovak-Soviet relations during late socialism, showing that these were often understood in triangulation with connections to other Warsaw Pact states. As a license-holder and a supplier of technical drawings and some spare parts, the Soviet Union could be at the receiving end of Czechoslovak producers’ complaints. However, Soviet generals acted as mediators when the Warsaw Pact countries collaborating on the T-72’s construction came into conflict.

Instead of a straightforward image of Soviet “dominance” and satellite state acquiescence, T-72 production ultimately reinforces Pál Germuska’s findings that, since the mid-1950s,

Central Europe was not . . . a region subject to total Soviet exploitation, but a group of states cooperating both with one another and the Soviet Union on the basis of mutual interest and which displayed increasing autonomy on the world stage as well.^102^

The license for the T-72 tank cost the Prague Politburo twenty-nine million rubles when purchased from the Soviet Union in the mid-1970s.^103^ The Politburo allocated the coordination of its production to ZŤS Martin. This decision further anchored Martin into an intellectual community and supply chains spanning the Eastern Bloc. Earlier tank-making had also relied on resources from beyond the town’s limits, but here, a new communications infrastructure was created to manage the increasingly complex back-and-forth required for this armored vehicle’s production. Arms factory staff were to work with partners in the Polish town of Gliwice to coordinate the creation of tank parts in East Germany, Hungary, Bulgaria, and Romania (the Czechoslovaks were to oversee deliveries from the first two of these, while the Poles were to take charge of supplies from the second two). They created a commission to oversee this whose working languages were Russian, Polish, Slovak, and Czech. The goal was for staff at ZŤS to assemble two hundred and fifty tanks annually, and their counterparts at Bumar in Gliwice to assemble the same number.^104^

The tanks were to constitute a flagship of Warsaw Pact cooperation, but their deployment took longer than anticipated. Historian Zuzana Zahradníčková notes that the biggest problems Czechoslovak producers experienced were with their Polish counterparts.^105^ The work of the commission established to solve production problems appears not always to have satisfied the parties involved who, on at least one occasion, turned to the Soviet Union to mediate their dispute.^106^ According to Štaigl and Turza, the Soviet Union also stepped in to provide parts when the Polish side did not.^107^

The Soviet Union could, at other times, generate problems itself. In 1985, the Czechoslovak People’s Army General Staff singled out the Soviet Union as being too slow to provide documentation on repairing the T-72.^108^ Suppliers around Czechoslovakia and the Warsaw Pact aggravated the problem by failing to supply spare parts, making only enough to construct the tanks, but no more.^109^ Some suppliers complained that T-72 production shored up inequalities already existing in the socialist bloc (Bulgaria protested that it played the unprofitable and undesirable role of “low-tech” part supplier here).^110^

Not all cooperation fostered by T-72 production was international. Tank manufacturing also fostered linkages at the regional and even the local level. Zahradníčková notes, for example, that when Martin was selected as the center of T-72 construction, nearby arms town Dubnica was delegated the creation of the tanks’ turrets and a range of casts and forged pieces for assembly in Martin. These contracts led to the building of new halls and the “modernization” of parts of the Dubnica factory, which was itself soldered into the ZŤS enterprise in a bureaucratic restructuring related to the tank’s production in 1978.^111^

If such large-scale production bound Dubnica and Martin into closer forms of cooperation with one another—and with similar arms towns in neighboring states—then the reliance on others that it engendered sparked drives toward increased autonomy on producers’ parts. Martin’s daughter concern, ZŤS Dubnica, ultimately sought in the 1980s to reduce its reliance on imported steel, installing its own steel-treatment works in a bid to seize control of each stage of tank turret production.^112^

T-72 production took place under Soviet license, but not completely according to Soviet specifications. Slovak technicians were first given two tank prototypes in 1977, which they called “Adam I and Adam II.” They took two pieces of one to examine each of its parts, and the second was for disassembling and practicing reassembling. Then, ZŤS engineers began to tinker with the tank’s original design. While parts needed to be interchangeable with those of any other T-72 on the battlefield, explained technician Vladimír Jančovič, translations necessarily needed to be made away from the “Soviet model” to accommodate differences in production process, as he and his colleagues in Martin used different, locally-manufactured machines to make these same tanks.^113^ Thus, different means needed to be taken to achieve the same ends. The Soviet side appeared to understand this: the only specification the license-holders made was that alterations had to be transmitted to Moscow according to the measurements laid out in the original, Soviet technical drawings.^114^

The production process was fairly dynamic, with *zlepšováky* (improvements) delegated to ZŤS departments. Improving the tank was supposed to be a bi-directional process. Production was also influenced, recalls Jančovič, by Soviet feedback on the tank’s performance gained through use in the Soviet-Afghan War of 1979–1989.^115^ The rollout of the T-72 represented a large-scale attempt at standardizing the Warsaw Pact’s armies. But it produced the opposite effect at the sites of its production. Difference served as a point of pride for some tank producers when they recall their experiences today. According to Jančovič:

Our tank turret, when it was cast well—you could see the difference between the Polish tank with the Polish turret, and the Slovak tank with the Slovak turret. You could see the difference . . .

*How?*

In the quality of the casting. Our casting was better, and the way the casting was finished was better.^116^

On the other hand, Jančovič suggested that the motors made in Gliwice were better than anything Martin engineers could produce, on account of the different international links maintained by Poland at the time: “In the 1980s, the Poles got out of the socialist area a bit, and so they got some technology from America.” The quality of these “American” motors meant the Slovak cooperation partners left their construction entirely to Bumar.^117^

This account presents a simultaneous pooling of resources—some spanning beyond the socialist bloc—and an impetus to differentiate oneself from precisely the constituencies with whom one pooled one’s resources. This insistence upon difference is articulated in Jančovič’s pride at the metallurgical knowledge and prowess developed locally in Martin. Such insistence upon one’s uniqueness aligns with the officially sanctioned rhetoric of socialist “competition” between deeply interlinked enterprises, and states, and perhaps constituted here a response to implicit concerns that one may not be so very different from one’s “competitors” at all. Outside of Martin, Czechoslovak Communist ministers did certainly worry that foreign leaders might turn to “brotherly” socialist states producing identical products, with the result that they decided against renegotiating the terms of deals that were less and less profitable, for example, with an increasingly indebted Libya by the late 1980s.^118^

When production of the T-72 tank wound down in the late 1980s, Czechoslovak officials judged that, far from having forged relationships among the countries of the Eastern Bloc, the tank had pointlessly duplicated their efforts.^119^ The competition which underpinned its production for Jančovič and characterized the relationship between its biggest producers around the region was posited to have been futile. Rather than bringing the best out of each other through training and ritualized encounter, Defense Ministry staff saw the competition that the tank’s production had engendered as a needless waste of each producer’s strengths.^120^

Jančovič would beg to differ. It is rare in personal accounts of one’s life to insist on the insignificance of the work one has done. The fond recollection of T-72 production permeating the oral histories cited above has certainly been shaped, moreover, by the fact these interviews were conducted after a long period of widespread unemployment in arms towns like Martin resulting from conversion away from military production. Nevertheless, the contrast between ministerial and vernacular views of tank production shows how the “customary production logics” of small communities are, to paraphrase Appadurai, “intimately tied to larger regimes of value defined by large scale polities” without, however, necessarily collapsing into them or becoming one and the same.^121^

## Conclusion

Scholars arguing that historians should not overstate the economic value of socialist globalization processes to national economies (including some contributors to this special section) make an important point.^122^ But this article shows that such processes impacted Czechoslovak Cold War arms towns like Martin disproportionately. We should research precisely these towns to examine the highly militarized nature of globalization in socialist Eastern Europe—following the studies of universities, political offices, and international organizations based in large urban centers that launched this promising research agenda.^123^

If this article has shifted our focus on where socialist globalization took place, then it has likewise contributed to arguments about when this process happened. It has traced such events to the decades before the 1970s, which are sometimes invoked as a starting point for a new wave of globalization in general, and in Eastern Europe in particular. Although it created new international supply chains and communications infrastructures, the production of T-72s, which began at that time, reworked international connections already built around know-how, military technology, and business transactions centering upon Martin for decades on account of its tank production. Perhaps counterintuitively, this new tank’s rollout also led to a redoubling of efforts to retrofit older models long since produced at the plant, as this increasingly became an economic imperative.

International linkages shaped production, producers, and the products created in Martin during the Cold War. The role of assembling finished tanks cemented Martin’s privileged position in relation to part-suppliers elsewhere in the Warsaw Pact. It also led ZŤS staff to produce more parts and raw materials in-house. Production shaped ZŤS employees’ solidarities, but also created rivalries, in ways captured by Vladimir Jančovič. The T-72 tanks produced in Martin were shaped by a lack of access to the original Soviet production machinery, but Soviet experience of combat in Afghanistan provided invaluable feedback for technicians who modified the tank accordingly. These three instances capture some of the frictions that shaped military globalization in Martin and other Cold War arms towns.

Socialist globalization’s different strands (military and civilian, cultural, political, and economic) cohabited in just such highly connected spaces. This can be illustrated using the analogy of the different economic tracks which ran in parallel with each other in Martin—economies in convertible currencies, transferrable rubles, Czechoslovak crowns, and barter, alongside a moral economy articulated by the employees of the ZŤS arms factory.

These inconvertible and non-convergent economies present in Cold War Martin did not negate each other. Instead, they give us a concrete and traceable example of the different tracks of internationalism and hyper-local networks of dependency and connection that shaped the interactions of inhabitants of this arms town during the Cold War. They show, moreover, the lumpiness, inconsistencies, and incongruities involved in socialist forms of globalization—and also the inequalities both within socialist states and the wider socialist world that such globalizing initiatives shored up. Ultimately, I suggest that Martin, a paradigmatic Czechoslovak arms town, was simultaneously open and closed—just like the arms plant that could not appear fully and yet put the town firmly on the map.

## Notes

1. Thank you to Frederick Corney, Mary Neuburger, Jannis Panagiotidis, and participants in Charles University’s Cold War Research Group seminar and the Czech Studies Workshop at University of Texas at Austin for their feedback on earlier drafts of this article. I am grateful also to Matěj Bílý, who generously shared archival materials that contributed to the arguments made here. Thank you to Réka Kinga Papp for telling me the joke that opens the article. Lastly, I thank the two anonymous peer reviewers and, above all, Réka Krizmanics and Béla Tomka for their persistence in bringing this special section to completion.

2. Arjun Appadurai, *The Social Life of Things: Commodities in Cultural Perspective* (Cambridge: Cambridge University Press, 1986), 30.

3. Oscar Sanchez-Sibony, *Red Globalization: The Political Economy of the Soviet Cold War from Stalin to Khrushchev* (Cambridge: Cambridge University Press, 2014), and James Mark, Paul Betts, Alena Alamgir, Péter Apor, Eric Burton, Bogdan C. Iacob, Steffi Marung, and Radina Vučetić, *Socialism Goes Global: The Soviet Union and Eastern Europe in the Age of Decolonization* (Oxford: Oxford University Press, 2022).

4. On the idea that the 1970s marked the beginning of a new wave of globalization, see Sebastian Conrad, *What Is Global History?* (Princeton, NJ: Princeton University Press, 2016), 97. For the significance of this decade anchoring Central and Eastern European economies into global markets, see Besnik Pula, *Globalization under and after Socialism: The Evolution of Transnational Capital in Central and Eastern Europe* (Stanford, CA: Stanford University Press, 2018).

5. Rostislav Králík, interview with the author, 30 April 2021; Miroslav Bero, interview with the author, 9 November 2023.

6. Goran Musić, *Making and Breaking the Yugoslav Working Class—The Story of Two Self-Managed Factories* (Budapest: Central European University Press, 2021); Anna Calori, *Engineering Global Socialism: Ownership, Non-Alignment, and Corporate Culture in a Bosnian Company* (Bloomington, IN: Indiana University Press, 2026).

7. Slovak National Archive, Bratislava, Slovak Republic. Fond ZŤS NOVA—Nová Dubnica, nos. 643, 660.

8. Teresa Da Silva Lopes, Christina Lubinski and Heidi J. S. Tworek, eds., *The Routledge Companion to the Makers of Global Business* (New York: Routledge, 2020), and Christopher Bayly, *The Birth of the Modern World, 1780-1914* (Hoboken, NJ: Wiley-Blackwell, 2003).

9. Michael Geyer and Adam Tooze, *The Cambridge History of the Second World War* (Cambridge: Cambridge University Press, 2015), vol. 3.

10. Felix Brahm and Daniel Stahl, “Arms Regimes across the Empires,” *Journal of Modern European History* 19, no. 4 (2021): 411–415.

11. Between 1981 and 1985. National Archive of the Czech Republic, Státní plánovácí komise, doc. 054124 “Přehled o vývoji struktury a proporcí výroby speciální techniky v ČSSR za období od 2. do 8. pětiletky,” 27 April 1988.

12. Paul Chamberlin, *The Cold War’s Killing Fields: Rethinking the Long Peace* (New York: Harper Collins, 2018).

13. Wolfgang Mueller and Peter Svik, eds., *Technological Innovation, Globalization and the Cold War: A Transnational* History (London: Routledge, 2022), 2.

14. Tarak Barkawi, *Globalization and War* (Lanham: Rowman & Littlefield, 2005), 61.

15. Ibid., 100.

16. The photos produced are often still classified, while the image analysis no longer is. American Central Intelligence Agency, file no. CIA-RDP82T00709R000102850001-1: “T-72 Tank Production, Martin, Czechoslovakia,” https://www.cia.gov/readingroom/docs/CIA-RDP82T00709R000102850001-1.pdf and file no. CIA-RDP78T04751A000700010015-2 “Weapons Testing Range Voroshilov (SKODA) Armament Plant Dubnica Nad Vahom, Czechoslovakia,” https://www.cia.gov/readingroom/docs/CIA-RDP78T04751A000700010015-2.pdf (both accessed 7 February 2024).

17. Angelika Epple, “Globalisierung/en,” Version: 1.0, in: *Docupedia-Zeitgeschichte*, 11 June 2012. http://docupedia.de/zg/Globalisierung (accessed 13 February 2026).

18. Barkawi, *Globalization and War*, 50.

19. Agata Zysiak, “‘Good’ and ‘Bad’ Workers and the Collapse of the Expected Life Course: The Postwar Working Class in Detroit (USA) and Łódź (Poland), 1940s-1980s,” *East European Politics and Societies* 35, no. 1 (2020): 3.

20. James Mark, Artemy Kalinovsky, and Steffi Marung, eds., *Alternative Globalizations: Eastern Europe and the Postcolonial World* (Bloomington, IN: Indiana University Press, 2020), 2.

21. Sanchez-Sibony, *Red Globalization*, 5–6.

22. National Archive of the Czech Republic, ÚV KSČ Antonín Novotný—zahraničí. Box 93—Egypt. “Насер обратился с просьбой о срочной поставке Египту . . .” dated 16 August 1956 and “Průběh projednávání dalších dodávek speciálního materiálu do SAR,” 14 October 1959.

23. On the motor vehicles made at ZŤS Martin, see Ján Okál’, Ivan Guráň, Jozef Baláž, and Juraj Šuhaj, “ZŤS - zlatá éra nášho strojárstva” in *MOTor*, 7 August 2020, 8–11.

24. Jan Štaigl and Peter Turza, “Zbrojná výroba na Slovensku v rokoch 1969-1992” (1. Časť) in *Vojenská História* 2 (2013): 101; Dušan Mihálik, “ANALÝZA: Efektivita zbrojárskej výroby na Slovensku za čias budovania socializmu,” *Armádní noviny*, 30 July. https://www.armadninoviny.cz/efektivita-zbrojarskej-vyroby-na-slovensku-za-cias-socializmu.html (accessed 4 April 2025).

25. Štaigl and Turza, “Zbrojná výroba na Slovensku,” 101–102; Mihálik, “ANALÝZA”

26. Béla Tomka, “Globalisation in Socialist Eastern Europe: A Turn in Research and Its Discontents,” *European History Quarterly* 53, no. 4 (2023): 692.

27. James Mark, Bogdan C. Iacob, Tobias Rupprecht, and Ljubica Spaskovska, *1989: A Global History of Eastern Europe* (Cambridge: Cambridge University Press, 2019), 51.

28. U.S. Arms Control and Disarmament Agency, “World Military Expenditures and Arms Transfers, 1990,” 102. https://2009-2017.state.gov/documents/organization/185650.pdf (accessed 13 February 2026).

29. Štaigl and Turza, “Zbrojná výroba na Slovensku,” 102.

30. Yudit Kiss, *Arms Industry Transformation and Integration: The Choices of East Central Europe* (Oxford: Oxford University Press, 2014), 258.

31. Figures calculated from František Ježek, *Prehľad výrobkov vozidlovej vojenskej techniky vyrábanej v Strojárskych závodoch Martin od roku 1952 do roku 1978. Popis a technické parametre* (Martin: ZŤS-VVÚ, 1995); František Ježek, *Výrobky vojenskej pásovej techniky v strojárenských závodoch v Martine v období 1981 až 2001*. Archív VÝVOJ, a. s. Martin, 2001 and Vladimír Francev, *Československé tankové síly, 1945-1992* (Prague: Grada, 2012). For the ZŤS production totals, see Francev, *Československé tankové síly*, 145 and 160–164.

32. Daniela Richterova, Mikuláš Pešta, and Natalia Telepneva, “Banking on Military Assistance: Czechoslovakia’s Struggle for Influence and Profit in the Third World, 1955-1968,” *The International History Review* 43, no. 1 (2020): 104, doi:10.1080/07075332.2020.1763422.

33. Pál Germuska, *Unified Military Industries of the Soviet Bloc: Hungary and the Division of Labor in Military Production* (Lanham, MD: Lexington Books, 2015), 27.

34. Ibid., 58.

35. Anonymous Omnipol employee. Interview with the author and Tomáš Nigrin, 3 April 2024.

36. Richterova et al., “Banking on Military Assistance,” 104; Germuska, *Unified Military Industries*, 165; Zuzana Zahradníčková, *“Vývoj výroby zbrojnej techniky a budovanie závodu v Dubnici nad Váhom v období rokov 1969-1990”* (MU Brno: magisterská diplomová práce, 2021), 108; Igor Baka, “Československá vojenská pomoc Líbyi v druhej polovici osemdesiatych rokov dvadsiateho storočia,” *Vojenská história* 16, no. 4 (2012): 25.

37. Germuska, *Unified Military Industries*, 151.

38. Anonymous Omnipol employee. Interview with the author and Tomáš Nigrin, 3 April 2024; Baka, “Československá vojenská pomoc Líbyi v druhej polovici osemdesiatych rokov,” 24.

39. Tomáš Nigrin, “Czechoslovakia’s arm trade (1948-1989) - sovereign or proxy actor?” paper presented at ESSHC, Leiden, 26 March 2025.

40. Security Forces Archive, Prague, File no. A 2-2 i.j. 453_f3270. “Pomoc frontu Národného oslobodenia Mozambiku,” 21 September 1965; File no. A 2-2 i.j. 474_f3320. “Správa o požiadavke Afrického ľudového zväzu ZIMBABWE o špeciálnu pomoc,” 14 October 1965.

41. Lenka Krátká, “‘Astounding It Is, what Lidice Carries’: The Transport of Czechoslovak Weapons for the Algerian National Liberation Front in 1959,” *International Journal of Maritime History* 33, no. 1 (2021): 54–69; Jan Koura, “Czechoslovakia and the ‘Cyprus Issue’ in the Years 1960–1974: Secret Arms Deals, Espionage, and the Cold War in the Middle East,” *Middle Eastern Studies* 57, no. 4 (2021): 516–33.

42. Nigrin, “Czechoslovakia’s Arm Trade.”

43. Richterova et al., “Banking on Military Assistance,” 95.

44. Petr Zídek, “Vývoz zbraní z Československa do zemí třetího světa v letech 1948-1962,” *Historie a Vojenství* 51, no. 3 (2002): 523–47; Lukacs Krajcsir, “Hungary and the Czech-Egyptian Arms Deal—Egypt’s Arms Purchases from Washington to Moscow,” in *Various Historian and Political Studies: Egypt & Hungary*, ed. Abdallah Abdel-Ati Al-Naggar (Cairo: Self-Published, 2019), 20–33.

45. Matyáš Borovský, “Czechoslovak Military Technical Assistance to Libya,” paper presented at the Charles University Cold War Research Seminar, 12 October 2022, 7.

46. Ibid.

47. Igor Baka, “Československá vojenská pomoc Líbyi v prvej polovici osemdesiatych rokov dvadsiateho storočia,” *Vojenská história* 16, no. 3 (2012): 80.

48. Ibid., 63.

49. Baka, “Československá vojenská pomoc Líbyi,” 65.

50. Central Intelligence Agency, “East European Involvement in the Gray Arms Market,” Document number CIA-RDP85T00283R000400070008-9, https://www.cia.gov/readingroom/docs/CIA-RDP85T00283R000400070008-9.pdf (accessed 10 April 2024).

51. Rosamund Johnston, “Věra Šťovíčková před mikrofonem - Rozhlas, politika a československo-africké vztahy v letech 1958–1968,” *Soudobé dějiny* 31, no. 1 (2024): 138–66.

52. Borovský, “Czechoslovak Military Technical Assistance to Libya,” 7.

53. Kiss, *Arms Industry Transformation and Integration*, 243.

54. National Archive of the Czech Republic, KSČ—Ústřední výbor 1945–1989, Praha—Předsedníctvo 1986–1989, P115/89, “Komplexní návrh postupu na efektivní využití volných kapacit výroby speciální techniky,” 2 June 1989.

55. Láník, “Vývoj čs. zbrojního průmyslu,” 93.

56. Ibid., 89, 94.

57. Zahradníčková, “Vývoj výroby zbrojnej techniky,” 72–73.

58. Central Intelligence Agency, file no. CIA-RDP80T00246A033200530001-4, “K. Y. Voroshilov Weapons Plant in Dubnica and Vahom,” 27 March 1957, K.Y. VOROSHILOV WEAPONS PLANT IN DUBNICA AND VAHOM (accessed 18 April 2025).

59. Mihálik, “ANALÝZA.”

60. Štátny archív v Žiline so sídlom v Bytči—pracovisko Archív Martin. OV KSS Martin Zapisnice Plena. “Referát,” 14 October 1954.

61. “Strojárne zmenili tvár mesta,” SME, 31 May 2018, https://myturiec.sme.sk/c/20838988/strojarne-zmenili-tvar-mesta.html (accessed 8 February 2024).

62. „Vzestup a pád slovenských zbrojovek,” *Hospodářské noviny*, 4 January 2013. https://archiv.hn.cz/c1-59061030-vzestup-a-pad-slovenskych-zbrojovek (accessed 10 April 2024).

63. Štátny archív v Žiline so sídlom v Bytči—pracovisko Archív Martin—okresna kontrolna & revizna komisia (OKRK). The case of J.Z., 4 July 1964.

64. Vladimír Jančovič, interview with the author, 18 May 2023.

65. Mihálik, “ANALÝZA.”

66. Pavel Kotrik, interview with the author, 14 February 2022.

67. Alena Alamgir, “Socialist Internationalism at Work: Changes in the Czechoslovak-Vietnamese Labor Exchange Program, 1967-1989,” (PhD diss., Rutgers University, 2014), 84.

68. Ibid., 21.

69. Slovak National Archive, Fond ZŤS NOVA—Nová Dubnica. No. 643, box 4. “Pokyny pro věcné, organizační a finanční zabezpečení odborné přípravy . . . vietnamských občanů . . .,” undated.

70. Slovak National Library, Tibor Buzák, “Pod stromčekom mieru,” *Turčiansky strojár* 28, no. 8 (23 February 1977): 7 and 31, no. 8 (25 February 1981): 3; on Chinese New Year as a holiday for Vietnamese workers, see Alamgir, “Socialist Internationalism at Work,” 97.

71. Pavel Kotrik, interview with the author, 14 February 2022; Vladimír Jančovič, interview with the author, 18 May 2023; Ján Segľa, interview with the author, 19 May 2023.

72. Baka, “Československá vojenská pomoc Líbyi v prvej polovici osemdesiatych rokov,” 75.

73. Borovský, “Czechoslovak Military Technical Assistance to Libya,” 13.

74. Baka, “Československá vojenská pomoc Líbyi,” 73.

75. Slovak National Library, “Vietnam,” *Turčiansky strojár* 29, nos. 11–12 (15 March 1968): 1.

76. Zdenka Hodická, interview with the author, 20 July 2020.

77. Slovak National Archive, Fond ZŤS NOVA—Nová Dubnica. No. 643, box 1. “Opatrenia na zvýšenú ochranu,” 20 February 1979.

78. Ibid.

79. Slovak National Archive, Fond ZŤS NOVA—Nová Dubnica. No. 643, box 6. “Zoznam utajovaných skutočností,” undated (in box of documents from 1983 to 1984).

80. Josef Mahovský, interview with the author, 23 June 2021; Pavel Kotrik, interview with the author, 14 February 2022; Vladimír Jančovič, interview with the author, 18 May 2023.

81. Mihálik, “ANALÝZA.”

82. Pavel Kotrik, interview with the author, 14 February 2022.

83. Slovak National Library, *Turčiansky strojár* 28, no. 2 (1978): 1

84. Ján Segla, interview with the author, 19 May 2023.

85. Kiss, *Arms Industry Transformation and Integration*, 262.

86. Slovak National Library, “Fragmenty z Mongolska,” *Turčiansky strojár* 31, no. 14 (8 April 1981): 3.

87. Appadurai, *The Social Life of Things*, 54.

88. Vladimír Ďuriš, interview with the author, 23 May 2023.

89. Slovak National Library, “Fragmenty z Mongolska,” *Turčiansky strojár* 31, no. 14 (8 April 1981): 3.

90. Slovak National Library, “Traktory do sveta,” *Turčiansky strojár* 28, no. 17 (20 April 1977): 1.

91. Slovak National Library, “S trstenským elkátečkom v Číne” *Turčiansky strojár* 35, no. 3 (23 January 1985): 6; “Kubánci doma,” *Turčiansky strojár* 19, no. 8 (22 February 1968): 1; “V krajine blízkych priateľov,” *Turčiansky strojár* 35, no. 4 (11 April 1985): 2.

92. Slovak National Library, “V exporte výrazné zlepšenie,” *Turčiansky strojár* 39, no. 4 (25 January 1989): 1.

93. Germuska, *Unified Military Industries*, 151.

94. Miroslav Belica, *Naši lidé v Kaddáfího Libyi—Nejen o zbraních, Semtexu a Lockerbie* (Prague: Epocha, 2014), 75.

95. Ibid.

96. Baka, “Československá vojenská pomoc Líbyi,” 44.

97. Vojenský ústřední archiv, box 59, no. 228, “Vyhodnocení plnění opatření z Plánů realizace protokolů,” 30 October 1985.

98. Ježek, *Prehľad výrobkov vozidlovej vojenskej*, 4.

99. Eva Hejduková, interview with the author, 22 May 2023.

100. Pavel Kotrik, interview with the author, 14 February 2022; Miroslav Bero, interview with the author, 11 November 2023.

101. Pavel Kotrik, interview with the author, 14 February 2022.

102. Germuska, *Unified Military Industries*, xvii.

103. Zahradníčková, “Vývoj výroby zbrojnej techniky,” 88.

104. National Archive of the Czech Republic, KSČ—Ústřední výbor 1945–1989, Praha—Předsedníctvo 1976–1981, P102/81, “Zpráva o sjednání dvoustranných mezivládních dohod o spolupráci a kooperaci při výrobě tanku T72,” 14 March 1979.

105. Zahradníčková, “Vývoj výroby zbrojnej techniky,” 82.

106. Ibid.

107. Štaigl and Turza, “Zbrojná výroba na Slovensku,” 94. On how the Polish partners viewed this cooperation, see Czesław Brózda, Zbigniew Bielec and Wojciech Gargała, *Bumar Pancerny: 55 lat Zakładów Mechanicznych Bumar-Łabędy S.A*. Gliwice: Zakłady Mechaniczne Bumar-tabędy S.A, 2006, 111–117.

108. Vojenský ústřední archiv box 59, no. 228, “Vyhodnoceni plneni opatreni z Planu realizace Protokolu vojenske rady Spojenych ozbrojenych sil,” 30 October 1985.

109. Ibid.

110. Matěj Bílý, *The Warsaw Pact, 1969-1985: The Pinnacle and Path to Dissolution* (Washington, DC: Academica Press, 2020).

111. Zahradníčková, “Vývoj výroby zbrojnej techniky,” 72–73.

112. Ibid., 78.

113. Vladimír Jančovič, interview with the author, 18 May 2023.

114. National Archive of the Czech Republic. KSČ—Ústřední výbor 1945–1989, Praha—Předsedníctvo 1986–1989, P115/89, “Komplexní návrh postupu na efektivní využití volných kapacit výroby speciální techniky,” 2 June 1989.

115. Vladimír Jančovič, interview with the author, 18 May 2023.

116. Ibid.

117. Ibid.

118. Zahradníčková, “Vývoj výroby zbrojnej techniky,” 108; Germuska, *Unified Military Industries of the Soviet Bloc*, 165; Baka, “Československá vojenská pomoc Líbyi,” 25.

119. Vojenský ústřední archiv, box 75, no. 292, “Vystoupeni predstavitele FMNO . . .” undated.

120. Ibid.

121. Appadurai, *The Social Life of Things*, 17.

122. Tomka, “Globalisation in Socialist Eastern Europe,” 693; Zsombor Bódy, “Globalization of Small Central European Countries after the Second World War: Hungary and Austria Compared” in this special section.

123. Marta Edith Holečková, *Příběh zapomenuté univerzity. Universita 17. listopadu (1961–1974) a její místo v československém vzdělávacím systému a společnosti* (Prague: Filozofická fakulta, 2019); Mikuláš Pešta, *Student Internationalism and the Global Cold War: The International Union of Students in Socialist Prague* (London: Bloomsbury Academic, 2026); Mark et al., *Alternative Globalizations*.

